# BMI-z score trajectories of Indonesian children and adolescents between 1993 and 2014 and associated risk factors

**DOI:** 10.1017/S1368980025100499

**Published:** 2025-06-03

**Authors:** Tri Nisa Widyastuti, Robin Turner, Helen Harcombe, Rachael McLean

**Affiliations:** Department of Preventive and Social Medicine, Dunedin School of Medicine, University of Otago, 18 Frederick Street, Dunedin 9016, New Zealand

**Keywords:** BMI-z score trajectory, Children and adolescents, Obesity, Indonesia

## Abstract

**Objectives:**

To identify trajectories of Indonesian children and adolescent’s BMI-z scores between 1993 and 2014, examine whether the pattern differs by sex and assess associations with host, agent and environmental factors.

**Design:**

Longitudinal data were from the Indonesian Family Life Survey with up to five measurements of height and weight. Group-based trajectory models investigated changes in BMI-z score across time; differences by sex were investigated using random effect (mixed) models. The association between the trajectories and host, agent and environmental factors were examined using multinomial logistic regression.

**Setting:**

Thirteen provinces in Indonesia.

**Participants:**

Indonesian children and adolescents aged 6–18 years (*n* 27 394 for BMI-z trajectories; *n* 8805 for risk factor analyses).

**Results:**

Mean BMI-z score increased from –0·743 sd in 1993 to –0·414 sd in 2014. Four distinct trajectory groups were estimated with mean BMI-z increasing more rapidly in the most recent time periods. One group (11·7 % of participants) had a mean BMI-z entirely within the moderately underweight range; two had trajectories in the normal range and one (5·6 %) had a mean BMI-z starting in the overweight range but within the obesity range by 2014. There were differences in trajectory groups by sex (*P*< 0·001). Those born in 2000s, frequent consumption of meat, fast foods, soft drinks and fried snacks, and living in urban areas were associated with rapid gain weight.

**Conclusions:**

These trajectories highlight the double burden of malnutrition and suggest that the prevalence of overweight and obesity is likely to increase substantially unless public health interventions are implemented.

Childhood obesity is increasing globally^(1)^. Obesity in childhood tracks to adulthood, with around 80–90 % of children living with obesity continuing to experience obesity as adults^(2)^. While childhood obesity remains a public health challenge in high-income countries, it has also become of increasing concern in low-middle-income countries (LMIC). It has been estimated that globally, approximately 205·5 million children and adolescents aged 5–19 years will be living with obesity by 2025^(1)^. Many LMIC that continue to have hunger and undernutrition are now also dealing with the existence of overnutrition in the community^(3)^. This situation creates a double burden of malnutrition and is associated with diet-related non-communicable diseases such as type 2 diabetes and CVD in later life^(4,5)^.

In Indonesia, evidence from repeated cross-sectional studies using the Indonesia National Basic Health Research Survey (INBHRS), the Indonesia Family Life Survey and the South East Asia Nutrition Survey (SEANUTS) shows that the overweight and obesity prevalence among Indonesian children and adolescents has increased over the last three decades^(6–10)^. Additionally, malnutrition remains prevalent in Indonesia creating a double burden^(11)^. The Indonesia Ministry of Health has adopted the WHO standards to be used among Indonesian children and adolescents, which supports the use of BMI z-scores for monitoring growth and weight status^(12)^. However, the majority of studies examining body weight have applied a cross-sectional or repeated cross-sectional design. Understanding the dynamic change or pattern of body weight and identifying risk factors early on have the potential to provide information that is crucial in developing prevention and intervention strategies. Longitudinal studies tracking BMI-z score trajectories alongside comprehensive assessments of host, agent and environment factors can provide valuable insights into the complex interplay of biological, behavioural and environmental influences on weight status over time. These factors are based on the classic epidemiological triad, a standard framework used in epidemiological analysis^(13)^.

This study aims to (1) describe the pattern of BMI-z scores across time, examining variations by age, survey wave and birth cohort, (2) identify distinct trajectories of BMI-z score of Indonesian children and adolescents from 1993 to 2014, (3) examine whether the trajectories of mean BMI-z score differs by sex and (4) describe the associations between host, agent, and environmental factors and BMI-z trajectory groups.

## Methods

### Study design and data sources

This study used publicly available data from the Indonesian Family Life Survey, a large population-based longitudinal study conducted over a 21-year period (1993–2014). The survey and sampling methods have been described in detail elsewhere^(14–18)^. Briefly, the survey involved five waves of data collection, in 1993, 1997, 2000, 2007 and 2014. The initial sampling frame included randomly selected households from thirteen out of twenty-seven provinces in Indonesia, representing around 83 % of the total population in 1993. The initial households along with their new additional family member/s due to marriage or birth were followed in the surveys conducted in waves 2–5. The anthropometry (weight and height) measures were taken by trained health workers^(18)^. A detailed description of the methods used for addressing aims 1–3, followed by the methods used for aim 4, is provided below.

#### Aims 1–3: Descriptive analysis and BMI-z score trajectory identification

Participants in this study included male and female children and adolescents aged 6–18 years. Observations where there were missing values on height or weight and from people who were pregnant at any wave were excluded (*n* 21 003). Implausible values of BMI for age (BMI-z score < –4 sd or > +5 sd) and height for age (HFA-z score < –5 sd or > +3 sd) were also excluded from the analysis (*n* 276)^(19)^. Individuals with missing values on sex, or where sex or age could not be verified from another wave, have also been excluded (*n* 47).

Weight status was mapped to age and sex-specific WHO 2007 BMI growth reference standards^(20)^. Steps for calculating BMI-z were as follows: (a) BMI was calculated as weight in kilograms (kg) divided by height in metres squared (m2) and (b) age-sex specific BMI-z score was calculated using zanthro package for Stata^(21)^. BMI-z score was analysed as a continuous variable. The WHO 2007 cut-points for underweight, overweight and obesity were used as a reference. The WHO 2007 growth reference defines BMI-z score (BMI-z) < −3 sd as severe underweight, ≥ −3 to < −2 sd as moderate underweight, ≥ −2 to < −1 sd as mild underweight, ≥ −1 to < +1 sd as normal weight, ≥ +1 sd to < 2 sd as overweight and ≥ 2 sd as obesity^(20,22)^.

Sex was categorised as male and female. Age was measured in months from the difference between the date of measurement and date of birth (taken from a birth certificate or birth records from physician/midwife). This was then categorised into childhood (6–11 years) or adolescence (12–18 years)^(23)^. Birth cohort was classified into decades of birth (1970s, 1980s, 1990s and 2000s).

### Statistical analysis

Sex, age and birth cohort were summarised using the number and percentage in each category based on the first measure per person (regardless of wave). BMI-z was summarised with mean and sd (by sex) and plotted in a histogram.

Descriptive plots were used to investigate the pattern of BMI-z across time including: (a) mean BMI-z by age for different survey waves, (b) mean BMI-z by survey wave for different ages and (c) mean BMI-z by age for different birth cohorts.

A group-based trajectory model was used to estimate the BMI-z trajectory groups using a censored normal distribution, utilising the statistical procedure ‘traj’ in Stata. We included all eligible participants who met the eligibility criteria for BMI-z score in the analysis. The number of groups was decided based on Bayesian information criterion, and the polynomial shape was chosen based on *P*-value < 0·05. Groups were constrained to have more than 5 % of the sample assigned to them^(24,25)^. Survey wave was used in the group-based trajectory model models to generate trajectories. Model fit was assessed by looking at the posterior probabilities and classification odds^(26)^. A sensitivity analysis was conducted where participants who had one measure only were excluded as they could not contribute information about the within-person change.

Mixed models, with random effects to account for the within person correlation, were used to test whether BMI-z score (outcome) differed by the identified trajectory groups sex (predictors)^(27,28)^. Specifically, mixed models were used to analyse the interaction between BMI trajectory groups and sex. The interaction with the birth cohort could not be assessed due to collinearity with the survey wave. Likelihood ratio *P*-values were used to assess the overall significance of variables and interaction terms. A sensitivity analysis was conducted using generalised estimating equations with a robust se to ensure any misspecification did not alter model results^(29)^.

#### Aim 4: Analysis of influencing factors on BMI-z trajectory groups

**Figure 1.**
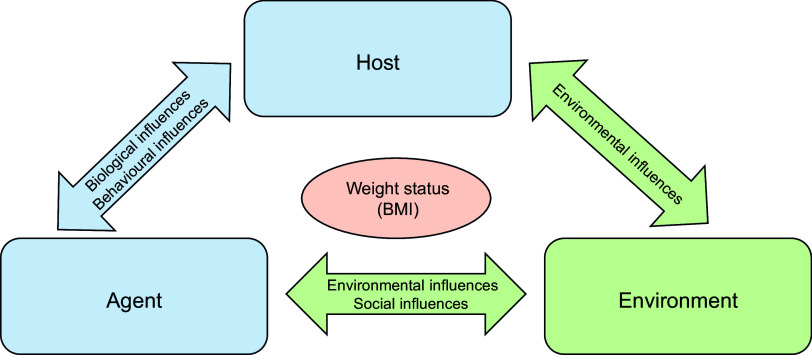
The conceptual framework used in the risk factors analysis adapted from the ‘Obesity ecological model’ by Egger and Swinburn.

To examine the relationship between various factors and BMI-z trajectory groups, we used the conceptual framework adapted from the obesity ecological model by Egger and Swinburn^(30)^ (Figure 1) to describe and analyse potential risk factors for overweight and obesity in Indonesian children and adolescents using relevant data available from the Indonesia Family Life Survey.

Host factors include biological factors such as age, sex, genetic and hormonal factors that influence body fatness^(30)^. In addition, the complex interaction of human behaviour that involves complex psychological aspects (emotions, thoughts and beliefs), individual habits, educational background, socio-economic status, family structure and parenting style are included as host factors^(30)^.

Agent refers to risk factors of overweight and obesity in terms of energy balance. Excessive intake of energy-dense foods and drinks such as high-fat foods, sugar-sweetened beverages and large portion sizes along with low energy expenditure such as low physical activity has been shown to promote overweight and obesity^(30)^.

Environmental factors include food supply factors and aspects of transport systems such as the availability of cycle tracks and pedestrian walkways. An ‘obesogenic environment’ is one where overweight and obesity can become prevalent^(30)^. Additionally, social influences such as social networks and social norms where they live and/or work may put an individual at risk of experiencing overweight or obesity^(31)^.

Host factor variables included sex, parental characteristics (parental education, parental employment, caregiver’s BMI) and total number of family members in the household (family size). Parental characteristics were based on the mother’s data when available with the father’s data used if mother’s data were unavailable in the Indonesia Family Life Survey dataset. Caregivers may be parents, or other relatives that are identified has having primary responsibility for the care of the child. Agent factor variables were derived from responses to a FFQ on meat, dairy, green leafy vegetables, fast food, soft drinks, fried snacks and sweet snacks. Residential area (rural/urban) was the environmental variable examined.

### Statistical analysis

Risk factor analysis was undertaken on a cross-sectional basis using 2014 data, as the FFQ data were available only in the 2014 survey. Trajectory group (based on classifying BMI-z scores from Aim 2) were used as an outcome in a cross-sectional analysis to understand which factors might be associated with membership of the different trajectory groups.

Multinomial logistic modelling essentially fits a logistic regression to each trajectory group *v*. a reference trajectory group. The model then gives an estimated multinomial OR. This represents the odds of being in that particular outcome trajectory group *v*. the reference trajectory group for a particular covariate comparison, similar to a traditional OR.

All analyses were conducted in Stata version 17.0^(32)^.

## Results

### Description and BMI-z score trajectories of children and adolescents

**Figure 2.**
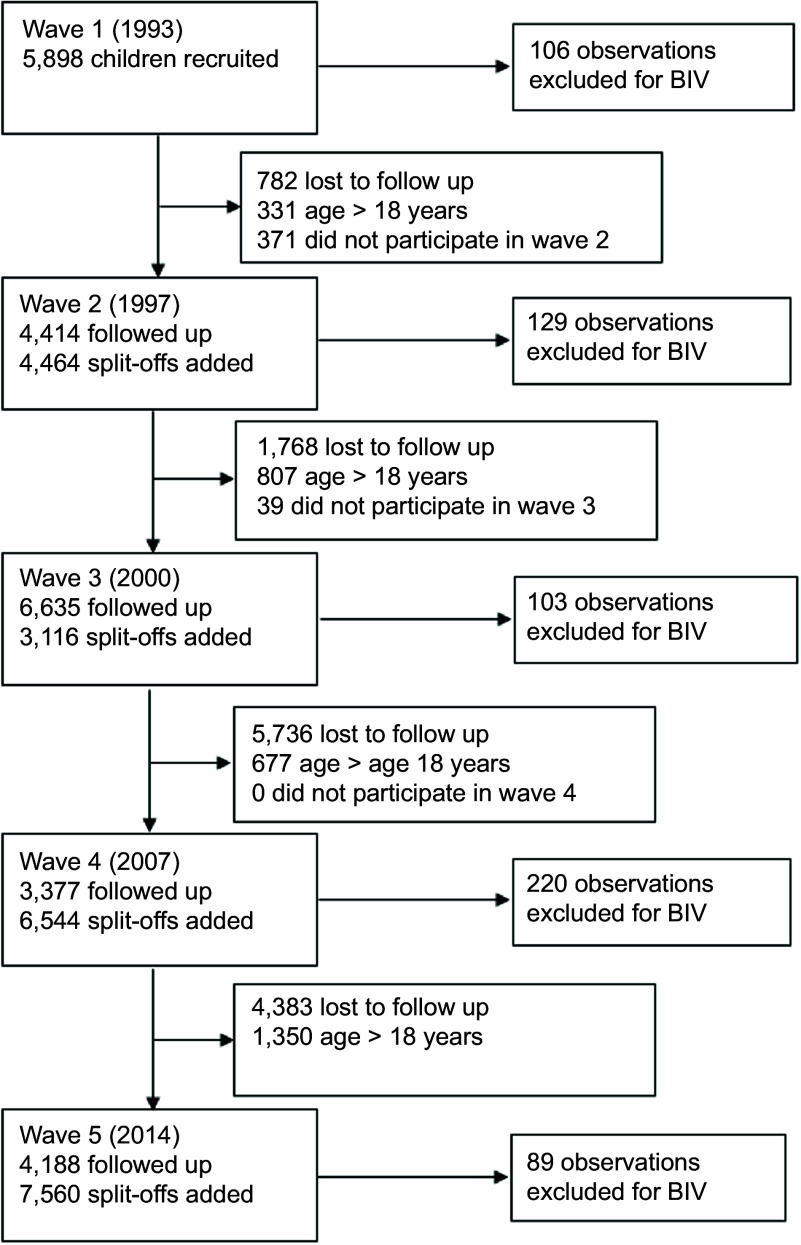
Flow diagram of exclusion criteria and total sample of Indonesian children and adolescents.

There were 45 549 observations from 27 394 children and adolescents included in the final analysis. Figure 2 shows the recruitment and exclusions across the study waves. The description of the included participants at first observation is presented in Table 1. The majority of participants (73·8 %) were aged 6–11 years when they first participated in the survey, followed by those aged 12–18 years (26·2 %). The proportion of males and females was approximately equal (50 % males, 50 % females). Only those born in the 1980s and 1990s could potentially participate in four survey waves; however, the maximum observed was three waves completed with 49·3 % of individuals participating in a single wave, 35·1 % in two and 15·6 % in three waves.

**Table 1.** Characteristics of the Indonesian children and adolescents participants at their first observations

Total	*n*	%
	27 394	100
Age group at recruitment		
Childhood (6–11 years)	20 220	73·8
Adolescence (12–18 years)	7174	26·2
Birth cohort		
1970s	1293	4·7
1980s	8658	31·6
1990s	8282	30·2
2000s	9161	33·5
Sex		
Male	13 705	50·0
Female	13 689	50·0
Number of waves completed		
1	13 509	49·3
2	9615	35·1
3	4270	15·6
BMI-z score	Mean	sd
Male	−0·64	1·26
Female	−0·55	1·14
	*n*	%
BMI-z score category		
Male		
Moderate underweight	1632	11·9
Mild underweight	3588	26·2
Normal weight	7254	52·9
Overweight	687	5·0
Obese	544	4·0
Female		
Moderate underweight	1251	9·1
Mild underweight	3252	23·8
Normal weight	8083	59·1
Overweight	766	5·6
Obese	337	2·5
BMI percentile category		
Male		
Underweight	2535	18·5
Healthy weight	9939	72·5
Overweight	460	3·4
Obesity	771	5·6
Female		
Underweight	2004	14·6
Healthy weight	10 582	77·3
Overweight	555	4·1
Obesity	548	4·0

Mean BMI-z score, which is measured in sd units, increased from –0·832 sd in 1993 to –0·471 sd in 2014 in males, and from –0·656 sd in 1993 to –0·355 sd in 2014 in females.

**Figure 3:**
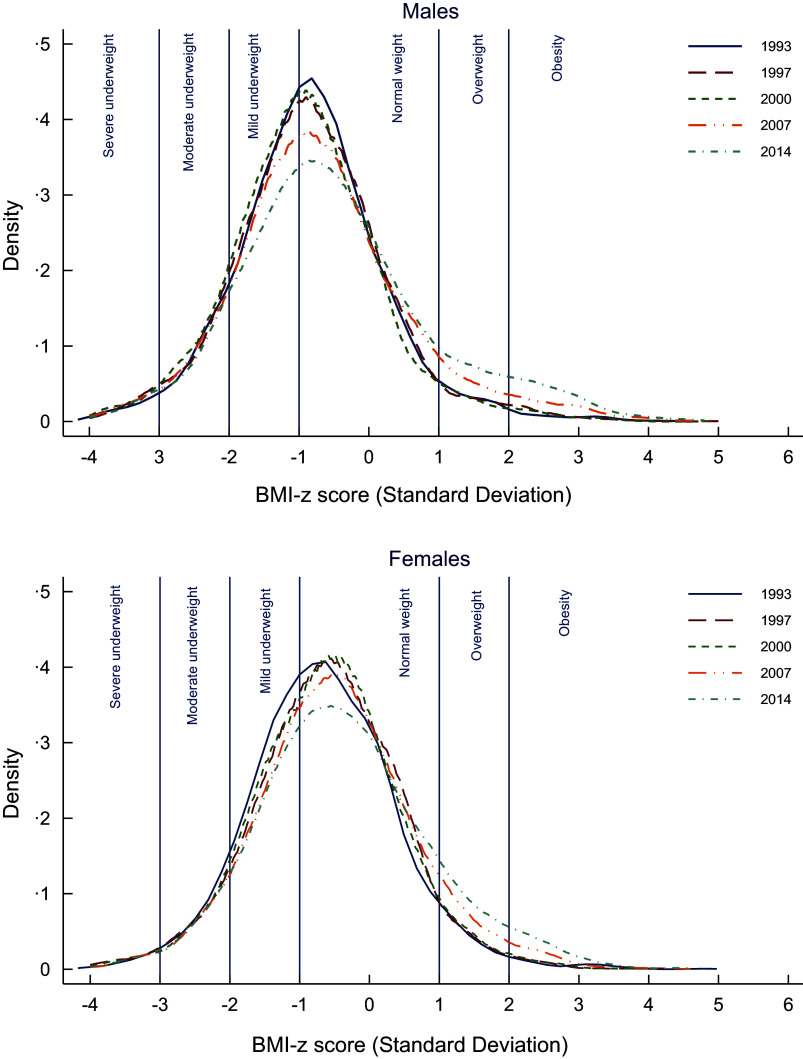
The distribution of BMI-z score in Indonesian children and adolescents, 1993–2014, separated by sex.

The prevalence of both overweight and obesity among male and female Indonesian children and adolescents increased considerably from 1993 to 2014 (Figure 3). Between 1993 and 2014, the prevalence of overweight increased almost two-fold in both males and females, increasing from 3·6 % to 7·5 % among males and 4·8 % to 9·4 % among females. The prevalence of obesity (using the WHO growth reference 2007) increased from 1·3 % to 7·0 % among males and from 1·3 % to 4·7 % among females.

**Figure 4.**
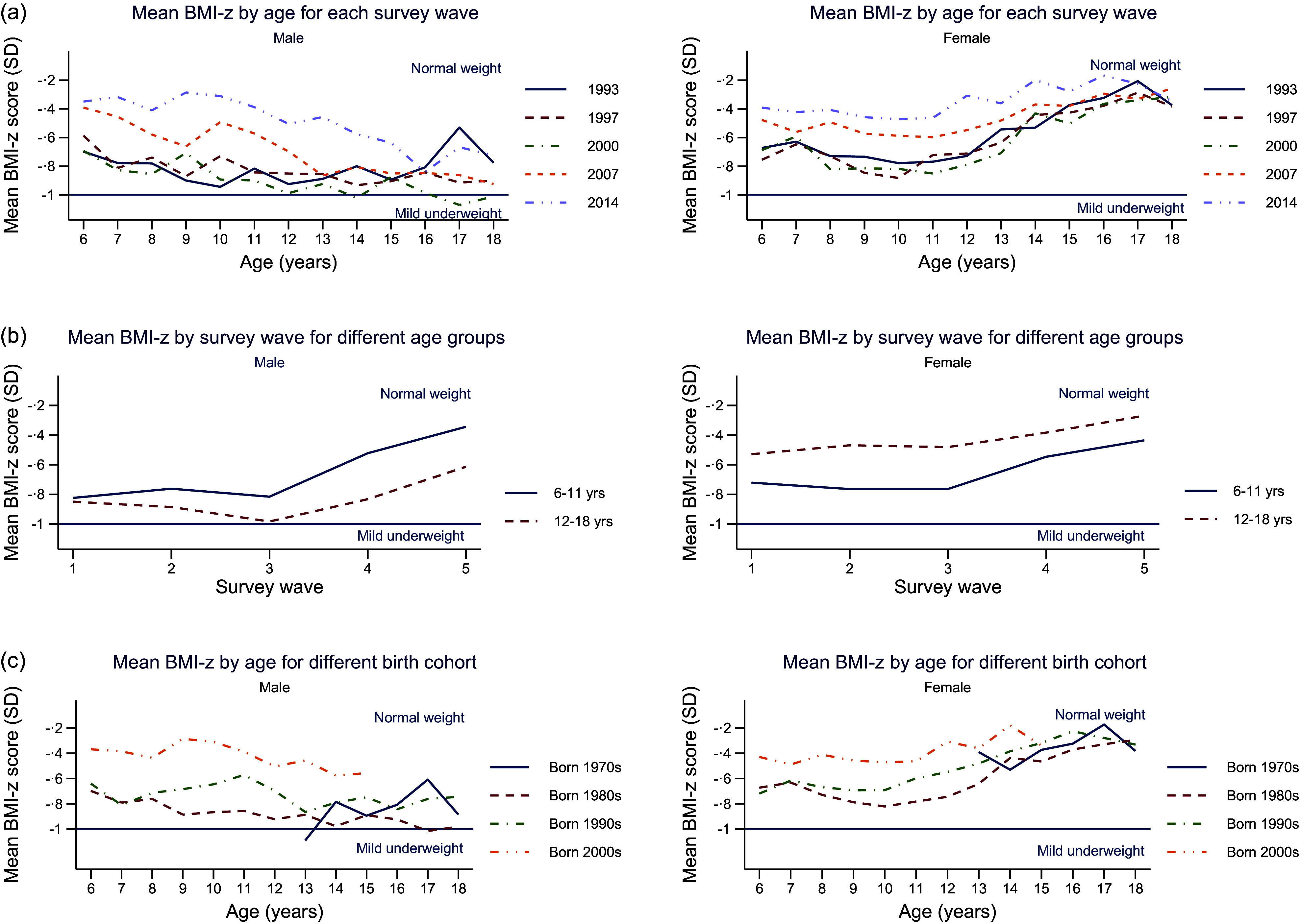
Mean BMI-z score of children and adolescents, separated by sex, showing variations across age groups, survey waves and birth cohorts.

Figure 4 shows mean BMI-z by age, year of survey and birth cohort stratified by sex. Figure 4(a) shows the mean BMI-z by age for each survey wave. Mean BMI-z decreased with age for males but increased with age for females in each survey wave. Mean BMI-z increased with each survey wave (1993–2014) for both males and females. Figure 4(b) shows mean BMI-z by survey wave for children and adolescents for males and females. Mean BMI-z generally increased over time from 1993 to 2014. In males, mean BMI-z was higher during childhood than during adolescence. In females, mean BMI-z was higher during adolescence than during childhood. Figure 4(c) shows the mean BMI-z by age for different birth cohorts. Mean BMI-z was higher overall for those born in the more recent birth cohort (2000) compared to those born in the earlier birth cohorts both for males and females.

**Figure 5.**
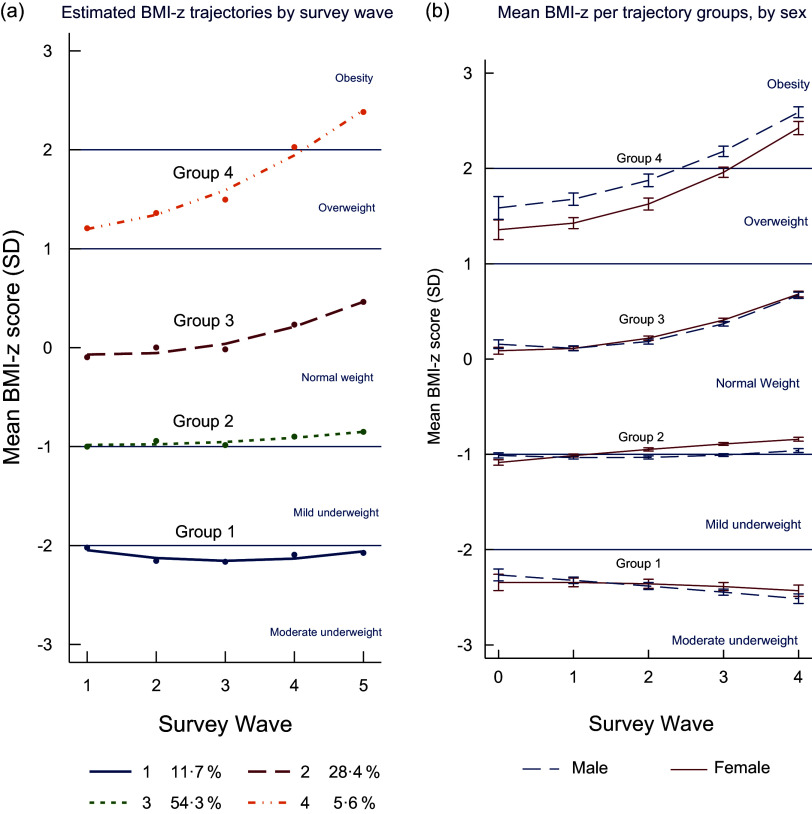
BMI-z trajectory groups of Indonesian children and adolescents.

Using the group-based trajectory model, four distinct trajectory groups were identified among Indonesian children and adolescents aged 6–18 years (Figure 5(a)). Groups 2, 3 and 4 were quadratic in shape, with an increasing slope in more recent waves. Group 1 (*n* 2344, 11·7 %) was characterised by the mean BMI-z being classified as moderately underweight throughout the period. Group 2, which included the majority of participants (*n* 16 503, 54·3 %), was characterised by having a mean BMI-z that was normal on average with a very slow increase over time. Group 3, which included just over a quarter of the participants (*n* 7151, 28·4 %), was characterised by the mean BMI-z being classified as normal throughout the period but with the mean BMI-z increasing faster than group 2. Group 4 had the smallest proportion of participants (*n* 1396, 5·6 %) and was characterised by the mean BMI-z being overweight at the beginning of the period and shifting to obesity at the end of the survey waves. There was a good model fit with an average posterior probability of assignment for each group higher than 0·7, the odds of correct classification were higher than 5·0 and the observed *v*. estimated proportion assigned to each group were similar (online Supplementary Material). Sensitivity analyses (excluding participants who had one measure only) found similar results to the main analyses.

Using mixed models, it was found that these BMI-z trajectory groups were different by sex (*P*< 0·001). Figure 5(b) shows the differences in mean BMI-z between males and females. The biggest difference is in group 4 with males having a higher mean BMI-z over time from 1993 to 2014 compared to females.

Sensitivity analyses using generalised estimating equations approach found similar results to the mixed model analyses.

### Risk factors of BMI-z score trajectories of children and adolescents

There were 8805 children and adolescents included in the cross-sectional analysis after 2851 individuals with any missing values in the dietary data were excluded. Table 2 presents the characteristics of participants by BMI-z trajectory groups for multinomial logistic regression. There were proportionately more children and adolescents born in the 2000s (7·1 %) in group 4 compared to those born in 1990s (3·4 %). Males had a higher proportion (8·6 %) in group 4 compared to females (5·5 %). Individuals who had parents with a university education had a higher proportion (11·3 %) in group 4 compared to those with parents who had no schooling (1·1 %). A greater proportion of participants with caregivers living with obesity (9·8 %) were in group 4 compared to those with underweight caregivers (3·0 %). Those having a family size of four or fewer (8·1 %) had a higher proportion in group 4 compared to a family size of more than four (6·2 %). Those who consumed meat, dairy, soft drinks, fried snacks and sweet snacks more than twice per week had a higher proportion in group 4 compared to those who did not consume these types of food/drink at all over the past week. A greater proportion of participants living in urban areas (9·1 %) were in group 4 than those living in rural areas (4·0 %).

**Table 2.** Characteristics of the 8805 children and adolescents included in cross-sectional analysis by trajectory groups, 2014

	BMI-z trajectory groups									
Variable	Group 1		Group 2		Group 3		Group 4		Total	
	*n* 667		*n* 5329		*n* 2182		*n* 627		*n* 8805	
	*n*	%	*n*	%	*n*	%	*n*	%	*n*	%
Host factors										
Birth cohort										
1990s	6	10·3	33	56·9	17	29·3	2	3·4	58	100
2000s	661	7·6	5296	60·5	2165	24·8	625	7·1	8747	100
Sex										
Female	313	7·3	2582	60·3	1151	26·9	237	5·5	4283	100
Male	354	7·8	2747	60·7	1031	22·8	390	8·6	4522	100
Parental education										
No school	26	13·9	118	63·1	41	21·9	2	1·1	187	100
Elementary	198	8·7	1483	64·8	495	21·6	112	4·9	2288	100
Junior/senior HS	344	7·1	2911	60·2	1238	25·6	345	7·1	4838	100
University	99	6·6	817	54·8	408	27·3	168	11·3	1492	100
Parental employment										
Not working	236	7·1	2000	60·2	843	25·4	242	7·3	3321	100
Working	431	7·9	3329	60·7	1339	24·4	385	7·0	5484	100
Caregiver’s BMI										
Underweight	78	18·1	288	66·8	52	12·1	13	3·0	431	100
Normal weight	261	10·2	1667	65·4	512	20·1	108	4·2	2548	100
Overweight	121	8·0	963	64·0	345	22·9	76	5·0	1505	100
Obese	191	4·7	2286	55·7	1225	29·8	404	9·8	4106	100
Missing	16	7·4	125	58·1	48	22·3	26	12·1	215	100
Family size										
≤ 4	297	7·0	2512	58·9	1113	26·1	346	8·1	4268	100
> 4	370	8·2	2817	62·1	1069	23·6	281	6·2	4537	100
Agent factors										
Food frequency (per week)										
Meat										
< 1 time	269	8·2	2121	64·3	734	22·3	173	5·2	3297	100
1–2 times	264	7·4	2128	59·7	917	25·7	256	7·2	3565	100
> 2 times	134	6·9	1080	55·6	531	27·3	198	10·2	1943	100
Dairy										
< 1 time	365	7·4	2996	60·8	1238	25·1	325	6·6	4924	100
1–2 times	102	7·9	805	62·2	305	23·6	82	6·3	1294	100
> 2 times	200	7·7	1528	59·1	639	24·7	220	8·5	2587	100
Green leafy vegetables										
< 1 time	180	7·5	1492	61·9	565	23·4	173	7·2	2410	100
1–2 times	160	7·1	1378	60·9	555	24·5	169	7·5	2262	100
> 2 times	327	7·9	2459	59·5	1062	25·7	285	6·9	4133	100
Fast food										
< 1 time	555	7·7	4424	61·3	1774	24·6	460	6·4	7213	100
1–2 times	82	7·3	608	54·4	293	26·2	134	12·0	1117	100
> 2 times	30	6·3	297	62·5	115	24·2	33	6·9	475	100
Soft drink										
< 1 time	552	7·7	4406	61·3	1735	24·1	497	6·9	7190	100
1–2 times	85	6·9	714	58·1	340	27·7	90	7·3	1229	100
> 2 times	30	7·8	209	54·1	107	27·7	40	10·4	386	100
Fried snack										
< 1 time	238	7·9	1835	61·0	743	24·7	192	6·4	3008	100
1–2 times	173	7·3	1410	59·1	609	25·5	194	8·1	2386	100
> 2 times	256	7·5	2084	61·1	830	24·3	241	7·1	3411	100
Sweet snack										
< 1 time	124	7·2	1040	60·2	460	26·6	105	6·1	1729	100
1–2 times	109	6·8	959	59·6	435	27·0	107	6·6	1610	100
> 2 times	434	7·9	3330	60·9	1287	23·5	415	7·6	5466	100
Environment factor										
Residence										
Rural	315	9·3	2199	64·9	741	21·9	135	4·0	3390	100
Urban	352	6·5	3130	57·8	1441	26·6	492	9·1	5415	100

The multinomial logistic regression (fully adjusted estimates) investigating the association between selected characteristics and BMI-z trajectory groups is presented in Table 3. The reference group was group 2.

**Table 3.** Multinomial logistic regression analysis investigating the associations between selected characteristics and BMI-z trajectory groups of Indonesian children and adolescents, 2014 on 8805 participants

Variable	Group 2 (Ref.) *n* 5329	Adjusted Model (*v*. Group 2)					
		Group 1 *n* 667		Group 3 *n* 2182		Group 4 *n* 627	
		Multinomial OR	95 % CI	Multinomial OR	95 % CI	Multinomial OR	95 % CI
Host factors							
Birth cohort							
1990s		Ref.	Ref.	Ref.	Ref.	Ref.	Ref.
2000s		0·64	0·26, 1·55	0·89	0·49, 1·61	2·25	0·53, 9·54
Sex							
Female		Ref.	Ref.	Ref.	Ref.	Ref.	Ref.
Male		1·07	0·91, 1·26	0·84	0·76, 0·93	1·56	1·31, 1·86
Parental education							
No school		Ref.	Ref.	Ref.	Ref.	Ref.	Ref.
Elementary		0·67	0·42, 1·05	0·90	0·62, 1·32	4·21	1·02, 17·42
Junior/senior HS		0·61	0·39, 0·96	1·08	0·74, 1·56	5·46	1·33, 22·44
University		0·62	0·38, 1·01	1·19	0·81, 1·76	8·03	1·94, 33·22
Parental employment							
Not working		Ref.	Ref.	Ref.	Ref.	Ref.	Ref.
Working		1·09	0·92, 1·29	0·95	0·86, 1·06	0·98	0·83, 1·17
Caregiver’s BMI							
Underweight		Ref.	Ref.	Ref.	Ref.	Ref.	Ref.
Normal weight		0·58	0·44, 0·77	1·69	1·23, 2·31	1·35	0·75, 2·45
Overweight		0·48	0·35, 0·65	1·90	1·38, 2·62	1·48	0·81, 2·72
Obese		0·31	0·23, 0·42	2·86	2·11, 3·88	3·41	1·93, 6·04
Missing		0·46	0·26, 0·82	2·14	1·37, 3·35	4·85	2·39, 9·87
Family size							
≤ 4		Ref.	Ref.	Ref.	Ref.	Ref.	Ref.
> 4		1·17	0·99, 1·38	0·81	0·73, 0·90	0·67	0·56, 0·80
Agent factors							
Food frequency (per week)							
Meat							
< 1 time		Ref.	Ref.	Ref.	Ref.	Ref.	Ref.
1–2 times		1·03	0·86, 1·25	1·16	1·03, 1·31	1·15	0·93, 1·42
> 2 times		1·05	0·83, 1·33	1·30	1·13, 1·50	1·54	1·22, 1·94
Dairy							
< 1 time		Ref.	Ref.	Ref.	Ref.	Ref.	Ref.
1–2 times		1·05	0·83, 1·33	0·88	0·76, 1·03	0·83	0·64, 1·08
> 2 times		1·14	0·94, 1·39	0·90	0·79, 1·01	0·92	0·76, 1·12
Green leafy vegetables							
< 1 time		Ref.	Ref.	Ref.	Ref.	Ref.	Ref.
1–2 times		0·99	0·79, 1·24	1·06	0·92, 1·22	1·09	0·86, 1·37
> 2 times		1·05	0·87, 1·28	1·20	1·06, 1·36	1·09	0·88, 1·34
Fast food							
< 1 time		Ref.	Ref.	Ref.	Ref.	Ref.	Ref.
1–2 times		1·18	0·92, 1·53	1·02	0·88, 1·20	1·52	1·22, 1·90
> 2 times		0·82	0·56, 1·22	0·90	0·72, 1·13	0·84	0·57, 1·24
Soft drink							
< 1 time		Ref.	Ref.	Ref.	Ref.	Ref.	Ref.
1–2 times		0·94	0·74, 1·21	1·17	1·01, 1·35	0·91	0·71, 1·16
> 2 times		1·16	0·78, 1·73	1·30	1·02, 1·66	1·58	1·10, 2·28
Fried snack							
< 1 time		Ref.	Ref.	Ref.	Ref.	Ref.	Ref.
1–2 times		0·99	0·80, 1·22	1·02	0·90, 1·16	1·20	0·97, 1·49
> 2 times		0·94	0·78, 1·14	0·98	0·87, 1·11	1·05	0·85, 1·29
Sweet snack							
< 1 time		Ref.	Ref.	Ref.	Ref.	Ref.	Ref.
1–2 times		0·98	0·74, 1·29	0·94	0·80, 1·11	0·93	0·70, 1·25
> 2 times		1·11	0·89, 1·38	0·79	0·70, 0·91	1·04	0·82, 1·31
Environment factor							
Residence							
Rural		Ref.	Ref.	Ref.	Ref.	Ref.	Ref.
Urban		0·82	0·69, 0·98	1·24	1·11, 1·38	1·98	1·60, 2·44

Of the host factors examined, those born in the 2000s compared to those born in the 1990s had 0·64 (95 % CI 0·26, 1·55) and 0·89 (95 % CI 0·49, 1·61) times the odds of being in group 1 and 3, but 2·25 (95 % CI 0·53, 9·54) times higher odds of being in group 4. Children and adolescents who had parents with a university education background compared to the reference group (parents with no schooling) had 0·62 (95 % CI 0·38, 1·01) times lower odds of being in group 1, while they had 1·19 (95 % CI 0·81, 1·76) and 8·03 (95 % CI 1·94, 33·22) times higher odds of being in group 3 and 4, respectively. Individuals with caregivers living with obesity compared to individuals with underweight caregivers had 0·31 (95 % CI 0·23, 0·42) times lower odds of being in group 1 while 2·86 (95 % CI 2·11, 3·88) and 3·41 (95 % CI 1·93, 6·04) times higher odds of being in groups 3 and 4. Having a family size of more than four compared to a family size of four or fewer had 1·17 (95 % CI 0·99, 1·38) times higher odds of being in group 1 while 0·81 (95 % CI 0·73, 0·90) and 0·67 (95 % CI 0·56, 0·80) times lower odds of being in groups 3 and 4. Being male compared to female had 1·07 (95 % CI 0·91, 1·26) and 1·56 (95 % CI 1·31, 1·86) times higher odds of being in groups 1 and 4, respectively, while 0·84 (95 % CI 0·76, 0·93) times lower odds of being in group 3.

Of agent factors examined, frequent consumption of meat (≥ 2 per week compared to > 1 per week) had 1·30 (95 % CI 1·13, 1·50) and 1·54 (95 % CI 1·22, 1·94) times higher odds of being in groups 3 and 4. Frequent consumption of fast food and soft drinks compared to less frequent consumption had 1·52 (95 % CI 1·22, 1·90) and 1·58 (95 % CI 1·10, 2·28) times higher odds, respectively, of being in group 4. Frequent consumption of fried snacks compared to less frequent consumption had 1·20 (95 % CI 0·97, 1·49) times higher odds of being in group 4.

In environmental factors, the odds of participants living in urban areas being in groups 3 and 4 were 1·24 (95 % CI 1·11, 1·38) and 1·98 (95 % CI 1·60, 2·44) times higher than participants in rural areas while 0·82 (95 % CI 0·69, 0·98) times lower odds of being in group 1.

## Discussion

This study clearly demonstrates the persistent double burden of malnutrition among Indonesian children and adolescents between 1993 and 2014. Mean BMI-z increased over the period, but was consistently below 0, indicating that Indonesian children were on average underweight compared to WHO international reference standards. Group-based trajectory modelling clearly demonstrates the double burden with group 1 (11·7 %) persistently underweight and group 4 (5·6 %) transitioned from overweight to obesity over the period (Figure 4(a)). Groups 2, 3 and 4 had quadratic increases in mean BMI-z over time, with mean BMI-z of groups 3 and 4 increasing more than group 2, while the greatest increase in mean BMI-z for groups 3 and 4 occurred between 2007 and 2014 (waves 4 and 5).

Another important finding from this study is that the mean BMI-z score for individuals in trajectory group 4 was overweight at the beginning of the survey but shifted to being obese at the last survey. Although only 5·6 % of the total sample was in this trajectory group, it is almost four million individuals when translated into actual numbers in the population of around 66 million Indonesian children and adolescents in 2017^(33)^. The fact that trajectory group 4 increased their mean BMI-z score much faster over time than those who started from healthy weight or underweight shows that there is a group of young people who are particularly vulnerable to nutrition-related non-communicable diseases in later life^(34)^. It is worth noting that the 1997–1998 Asian financial crisis resulted in Indonesia experiencing a substantial downturn in economic growth, with around 36 million additional individuals falling into absolute poverty^(35)^. Indonesia gained its positive economic growth from 2000^(36)^, which may explain why BMI-z increased more rapidly in children and adolescents in more recent waves.

This study shows that those born in 2000s, those who had parents with a university education background, had caregiver with obesity, living in urban area, frequent consumption of meat, fast food, soft drinks and fried snacks were found to be more likely to belong to group 4, the trajectory characterised by the mean BMI-z score being overweight at the beginning of period and shifting to obesity at the end of the survey.

This is the first study examining trends of BMI among Indonesian children and adolescents using BMI for age z score as a continuous variable. Previous studies treated BMI-z score as a categorical variable and used different classifications to define overweight and obesity (WHO 1977, IOTF 2000, CDC 2000 and WHO 2007), resulting in inconsistency in the prevalence reported across studies^(37)^. Our analysis using the WHO 2007 growth reference showed that in 2014, 7·5 % of males and 9·4 % of females were overweight and 7·0 % of males and 4·7 % of females aged 6–18 years were classified as obese. Many High-Income Countries (as defined by World Bank) have a higher prevalence of overweight and obesity. For example, the prevalence of obesity among children and adolescents aged 2–19 years in the USA using data from the National Health and Nutrition Examination Survey (NHANES) between 2017 and 2020 was 20·9 % for boys and 18·5 % for girls^(38)^. The Global Burden of Disease study showed that in 2013 23·8 % of boys and 22·6 % of girls aged 2–18 years were classified as overweight or living with obesity using International Obesity Task Force (IOTF) cut-offs in developed countries^(39)^. However, the trajectory analysis in this study demonstrates that the prevalence of overweight and obesity is likely to increase substantially over the next decade unless public health interventions are implemented.

The findings of the current study are similar to those from repeated cross-sectional analyses of body weight from other LMIC in the Asia region (Central, South, Southeast and East Asia), Africa region (North, West, Central, East and Southern Africa), Latin America countries, as well as Caribbean, Melanesia, Polynesia and Micronesia countries^(22,40)^. The Global Burden of Disease Study also reported an increase in the prevalence of overweight and obesity among children and adolescents in LMIC of around 4·8 % in boys and 5 % in girls between 1980 and 2013^(39)^. In the category of ‘Asian countries,’ it was estimated that the prevalence of overweight and obesity was higher among boys than girls^(41)^. These studies show that although the prevalence of obesity and overweight among children and adolescents is generally higher in HIC, overweight and obesity prevalence is increasing in LMIC, especially among those experiencing rapid economic development^(42)^. Recent comparative studies suggest that Indonesia has the highest annual increase in the prevalence of overweight and obesity among East Asia and Pacific countries^(4,5)^.

Studies examining trajectories of BMI using longitudinal study design in LMIC settings are limited. Two studies have been published from MIC: China and Iran. A study in China based on longitudinal data identified four (normal-stable, low normal-normal-stable, overweight-obese and low normal-normal-overweight) trajectory patterns of BMI over the life course (aged 6–60 years at baseline, consisting of seven waves from 1989–2009) with the quadratic effect among low normal to normal (majority of participants) and overweight to obese group (minority of participants)^(43)^. Another study based on a large-scale population-based prospective cohort study among Iranian adolescents identified two distinct BMI trajectories in males (normal weight and overweight-late obese) and three BMI trajectories in females (normal weight, overweight-early obese and overweight)^(44)^. Several studies have examined BMI trajectories using longitudinal data in HIC. A study from the International Childhood Cardio-vascular Cohort (i3C) Consortium, a consortium of countries from three HIC (Australia, Finland and the USA), identified five consistent BMI trajectory groups from childhood to mid-adulthood, with one BMI trajectory group (around 50 % of participants) maintaining a persistently low trajectory and the other four trajectory groups combined (50 % of participants) showing improving/progressing to high/very high BMI trajectories^(45)^. A study in the UK using the 1970 British Cohort Study (born in April 1970 at baseline, followed up in 1975, 1980, 1986, 1996, 2000, 2004, 2008 and 2012 at age 42 years) identified three latent class BMI trajectories; the first trajectory (92 %) was characterised by normal weight initially but gradually increasing; the second trajectory (4 %) was characterised by being persistently classified as having high BMI over time during childhood to young adulthood, the third trajectory (4 %) was characterised by starting with a normal weight during childhood but increasing exponentially during adolescence and young adulthood^(46)^. All of these studies identified BMI trajectory patterns that started from the normal weight range of BMI.

The ecological model of obesity risk factors proposed by Egger and Swinburn^(30)^ suggests that multiple levels of influence, including individual, interpersonal, community and societal factors, contribute to obesity development. These factors interact dynamically, shaping behaviours and outcomes related to weight gain and obesity^(47)^.

Childhood nutrition such as poor dietary habits (e.g. consumption of energy-dense, nutrient-poor foods such as sugary beverages and processed snacks) can influence long-term weight status and metabolic health^(48)^. Built environment characteristics, such as neighbourhood walkability, access to recreational facilities, availability of healthy food options and exposure to advertising can influence dietary behaviours and physical activity levels that impact obesity risk^(30)^.

Prevention and intervention programmes targeting both underweight and overnutrition are urgent^(4,42)^. Addressing this double burden of nutrition in Indonesia is vital and aligns with several international frameworks. Reducing malnutrition (both underweight and overweight) is a key component of Sustainable Development Goal 2 (zero hunger), with the target of reducing wasting and overweight among children under 5 years of age identified by UNICEF as an important child-related indicator^(49)^. The WHO has called on member states to halt the rise in obesity as one of nine key targets for reducing non-communicable diseases in its Global Action Plan^(50)^. Additionally, the WHO ECHO report on ‘Ending Childhood Obesity’ recommends several strategies, including promoting healthy diets, increasing physical activity and improving healthcare access^(51)^. This study shows that Indonesia is not making sufficient progress in this area, highlighting the need for urgent action to implement these strategies.

This is the first study observing trends of BMI-z in Indonesian children and adolescents examining different patterns of trajectories by sex and birth cohort. Strengths of the current study include the use of longitudinal data from a large population-based longitudinal survey covering a 21-year period, with more than 90 % re-contact rates at each wave at the household level^(18)^. An additional strength is that height and weight were measured using standard protocols by trained health workers, rather than by self-report. Adopting the theoretical framework of an ecological model proposed by Egger and Swinburn^(30)^ was also a strength as it helped to better explain the aetiology of overweight and obesity not only as an energy-balance equation but also through identifying the broader environmental factors that influence the energy balance of individuals^(52)^. There were however some limitations of the study, not all individuals participated in every wave they were eligible for, leading to some missing data and loss to follow-up^(28)^. However, this issue was minimised by using mixed model analysis. This model allowed the inclusion of all eligible participants regardless of how many times they participated in the study^(27,28)^. Additionally, dietary data was based on FFQ so it did not give precise measures of macronutrient intakes. However, FFQ are often used to estimate common intakes of foods and food groups in individuals^(53)^.

In conclusion, this study provides robust new evidence on the trajectories of body size among Indonesian children and adolescents. The mean BMI-z patterns confirm that Indonesia is in the process of a rapid and intense nutrition transition and is facing a double burden of malnutrition as a consequence^(5)^. While the majority of participants aged 6–18 years show increased BMI-z over the period, undernutrition persists in a small group. The disparity between those who are underweight and those who are overweight is greater among the youngest generation (born in the 2000s), the age group born after the Indonesian major financial crisis in 1997, suggesting that inequality rose markedly after this event^(54)^. Moreover, the potential implications of the positive quadratic effect (with greater increases in mean BMI-z over the period from the last three surveys in groups 2–4) indicates an accelerated increase in BMI-z that will likely translate to chronic health risk and increased non-communicable disease in adulthood and with a potential impact on public health and health services into the future. In addition, by analysing data using group-based trajectory models, researchers can identify how selected risk factors interact and contribute to different trajectories of overweight and obesity, informing targeted interventions and policies to prevent and manage weight-related health problems.

Further research using the same and/or other datasets to investigate childhood obesity and the health outcomes in adulthood is needed to continue to gain a better understanding of the epidemiology of overweight and obesity among Indonesian children and adolescents. This knowledge helps in informing and formulating targeted prevention and intervention efforts in the Indonesian child and adolescent population.

## Supporting information

Widyastuti et al. supplementary material Widyastuti et al. supplementary material

## Supplementary material

For supplementary material accompanying this paper visit https://doi.org/10.1017/S1368980025100499

## References

[1] World Obesity Federation (2020) Obesity: Missing the 2025 Global Targets – Trends, Costs and Country Reports. https://data.worldobesity.org/publications/WOF-Missing-the-2025-Global-Targets-Report-FINAL-WEB.pdf (accessed 4 June 2022).

[2] Ryder JR , Jacobs DR , Sinaiko AR et al. (2019) Longitudinal changes in weight status from childhood and adolescence to adulthood. *J Pediatr* 214, 187–192.e2. 10.1016/j.jpeds.2019.07.035 31493910

[3] Hawkes C , Ruel MT , Salm L et al. (2020) Double-duty actions: seizing programme and policy opportunities to address malnutrition in all its forms. Lancet 395, 142–155. 31852603 10.1016/S0140-6736(19)32506-1

[4] Popkin BM , Corvalan C & Grummer-Strawn LM (2020) Dynamics of the double burden of malnutrition and the changing nutrition reality. Lancet 395, 65–74. 31852602 10.1016/S0140-6736(19)32497-3 PMC7179702

[5] Popkin BM & Ng SW (2022) The nutrition transition to a stage of high obesity and noncommunicable disease prevalence dominated by ultra-processed foods is not inevitable. Obes Rev 23, e13366. 34632692 10.1111/obr.13366 PMC8639733

[6] Agustina R, Meilianawati, Fenny et al. (2021) Psychosocial, eating behavior, and lifestyle factors influencing overweight and obesity in adolescents. Food Nutr Bull 42, S72–S91. 34282658 10.1177/0379572121992750

[7] Kunto YS & Bras H (2021) Sibling inequalities in overweight and the role of mother’s education: evidence from the Indonesian family life survey. Food Nutr Bull 42, S21–S38.

[8] Nurwanti E , Hadi H , Chang JS et al. (2019) Rural-urban differences in dietary behavior and obesity: results of the riskesdas study in 10–18-year-old Indonesian children and adolescents. Nutrients 11, 2813. 31752101 10.3390/nu11112813 PMC6893820

[9] Oddo VM , Maehara M & Rah JH (2019) Overweight in Indonesia: an observational study of trends and risk factors among adults and children. BMJ open 9, e031198. 10.1136/bmjopen-2019-031198 PMC6773342 31562157

[10] Harahap H , Sandjaja S , Soekatri M et al. (2018) Association of energy intake and physical activity with overweight among Indonesian children 6–12 years of age. Asia Pac J Clin Nutr 27, 211–216. 29222901 10.6133/apjcn.032017.05

[11] Maehara M , Rah JH , Roshita A et al. (2019) Patterns and risk factors of double burden of malnutrition among adolescent girls and boys in Indonesia. PLOS ONE 14, e0221273. 31430324 10.1371/journal.pone.0221273 PMC6701791

[12] Indonesia Ministry of Health (2007) *National Report of Indonesia Basic Health Research 2007–2008* (Indonesia MoH, editor). Jakarta: Agency of Health Research and Development – Indonesia Ministry of Health.

[13] Mattsson M , Murray DM , Hawkes CP et al. (2021) Body Mass Index trajectories in the first 5 years and associated antenatal factors. Front Pediatr 9, 622381. 33681100 10.3389/fped.2021.622381 PMC7933027

[14] Frankenberg E , Karoly L , Gertler P et al. (1995) *The 1993 Indonesian Family Life Survey: Overview, Field Report*. https://www.rand.org/content/dam/rand/pubs/drafts/2007/DRU1195.1.pdf (accessed 3 February 2021).

[15] Frankenberg E & Thomas D (2000) *The Indonesia Family Life Survey (IFLS): Study Design and Results from Waves 1 and 2*. March; 2000. Santa Monica, CA: RAND Corporation.

[16] Strauss J , Beegle K , Sikoki B et al. (2004) *The Third Wave of the Indonesia Family Life Survey (IFLS3): Overview, Field Report. NIA/NICHD*. Santa Monica, CA: RAND Corporation.

[17] Strauss J , Witoelar F , Sikoki B et al. (2009) *The Fourth Wave of the Indonesian Family Life Survey (IFLS4): Overview, Field Report*. Santa Monica, CA: RAND Corporation.

[18] Strauss J , Witoelar F & Sikoki B (2016) *The Fifth Wave of the Indonesia Family Life Survey: Overview and Field Report*. Santa Monica, CA: RAND.

[19] Freedman DS , Lawman HG , Skinner AC et al. (2015) Validity of the WHO cutoffs for biologically implausible values of weight, height, and BMI in children and adolescents in NHANES from 1999 through 2012. Am J Clin Nutr 102, 1000–1006. 10.3945/ajcn.115.115576 PMC4631693 26377160

[20] de Onis M , Onyango AW , Borghi E et al. (2007) Development of a WHO growth reference for school-aged children and adolescents. Bull World Health Organ 85, 660–667. 18026621 10.2471/BLT.07.043497 PMC2636412

[21] Vidmar SI , Cole TJ & Pan H (2013) Standardizing anthropometric measures in children and adolescents with functions for Egen: update. Stata J 13, 366–378.

[22] Abarca-Gómez L , Abdeen ZA , Hamid ZA et al. (2017) Worldwide trends in body-mass index, underweight, overweight, and obesity from 1975 to 2016: a pooled analysis of 2416 population-based measurement studies in 128·9 million children, adolescents, and adults. Lancet 390, 2627–2642. 29029897 10.1016/S0140-6736(17)32129-3 PMC5735219

[23] Williams K , Thomson D , Seto I et al. (2012) Standard 6: age groups for pediatric trials. Pediatr 129, S153–S60. 10.1542/peds.2012-0055I 22661762

[24] Nagin DS (1999) Analyzing developmental trajectories: a semiparametric, group-based approach. Psychol Methods 4, 139. 10.1037/1082-989x.6.1.18 11285809

[25] Jones BL & Nagin DS (2013) A note on a Stata Plugin for estimating group-based trajectory models. Sociol Meth Res 42, 608–613.

[26] Nagin DS & Odgers CL (2010) Group-based trajectory modeling in clinical research. Ann Rev Clin Psychol 6, 109–138. 20192788 10.1146/annurev.clinpsy.121208.131413

[27] Diggle P (2002) Analysis of Longitudinal Data. Oxford: Oxford University Press.

[28] Singer JD & Willett JB (2003) Applied Longitudinal Data Analysis: Modeling Change and Event Occurrence. Oxford: Oxford University Press.

[29] Hubbard AE , Ahern J , Fleischer NL et al. (2010) To GEE or Not to GEE: comparing population average and mixed models for estimating the associations between neighborhood risk factors and health. Epidemiology 21, 467–474. 20220526 10.1097/EDE.0b013e3181caeb90

[30] Garry E & Boyd S (1997) An ‘ecological’ approach to the obesity pandemic. BMJ 315, 477. 9284671 10.1136/bmj.315.7106.477 PMC2127317

[31] Narciso J , Silva AJ , Rodrigues V et al. (2019) Behavioral, contextual and biological factors associated with obesity during adolescence: a systematic review. PLOS ONE 14, e0214941. 30958850 10.1371/journal.pone.0214941 PMC6453458

[32] StataCorp (2021) Stata Statistical Software: Release 17. College Station, TX: StataCorp LLC.

[33] Indonesia Ministry of National Development Planning (2017) SDG Baseline Report on Children in Indonesia. BAPPENAS and UNICEF. Jakarta: Ministry of National Development Planning.

[34] Song M (2019) Trajectory analysis in obesity epidemiology: a promising life course approach. Curr Opin Endocr Metab Res 4, 37–41. 30906899 10.1016/j.coemr.2018.08.002 PMC6426320

[35] Suryahadi A , Sumarto S & Pritchett L (2003) Evolution of poverty during the crisis in Indonesia. Asian Econ J 17, 221–241.

[36] Harvie C (1999) *Indonesia: Recovery from Economic and Social Collapse*. Wollongong, NSW: University of Wollongong, Department of Economics.

[37] Rachmi CN , Li M & Alison Baur L (2017) Overweight and obesity in Indonesia: prevalence and risk factors—a literature review. Public Health 147, 20–29. 28404492 10.1016/j.puhe.2017.02.002

[38] Stierman B , Afful J , Carroll MD et al. (2021) National Health and Nutrition Examination Survey 2017–March 2020 prepandemic data files development of files and prevalence estimates for selected health outcomes. *Natl Health Stat Rep* 158, 10–5620. 10.15620/cdc:106273 PMC11513744 39380201

[39] Ng M , Fleming T , Robinson M et al. (2014) Global, regional, and national prevalence of overweight and obesity in children and adults during 1980–2013; 2013: a systematic analysis for the Global Burden of Disease Study 2013. *Lancet* 384, 766–781. 10.1016/S0140-6736(14)60460-8 PMC4624264 24880830

[40] Di Cesare M , Sorić M , Bovet P et al. (2019) The epidemiological burden of obesity in childhood: a worldwide epidemic requiring urgent action. BMC Med 17, 212. 31760948 10.1186/s12916-019-1449-8 PMC6876113

[41] Mazidi M , Banach M & Kengne AP (2018) Prevalence of childhood and adolescent overweight and obesity in Asian countries: a systematic review and meta-analysis. Arch Med Sci 14, 1185–1203. 30393474 10.5114/aoms.2018.79001 PMC6209725

[42] Seferidi P , Hone T , Duran AC et al. (2022) Global inequalities in the double burden of malnutrition and associations with globalisation: a multilevel analysis of Demographic and Health Surveys from 55 low-income and middle-income countries, 1992–2013; 2018. *Lancet Global Health* 10, e482–e490. 10.1016/S2214-109X(21)00594-5 PMC8924053 35148831

[43] Islam MT , Möller J , Zhou X et al. (2019) Life-course trajectories of body mass index and subsequent cardiovascular risk among Chinese population. PLOS ONE 14, e0223778. 31600353 10.1371/journal.pone.0223778 PMC6786833

[44] Ahanchi NS , Ramezankhani A , Munthali RJ et al. (2019) Body mass index trajectories from adolescent to young adult for incident high blood pressure and high plasma glucose. PLOS ONE 14, e0213828. 31042715 10.1371/journal.pone.0213828 PMC6493705

[45] Cleland V , Tian J , Buscot M-J et al. (2022) Body-mass index trajectories from childhood to mid-adulthood and their sociodemographic predictors: evidence from the International Childhood Cardiovascular Cohort (i3C) Consortium. *eClinicalMedicine* 48, 101440. 10.1016/j.eclinm.2022.101440 PMC9112099 35706485

[46] Viner RM , Costa S & Johnson W (2019) Patterns of BMI development between 10 and 42 years of age and their determinants in the 1970 British Cohort Study. J Epidemiol Community Health 73, 79–85. 30409921 10.1136/jech-2018-211051

[47] Swinburn BA , Kraak VI , Allender S et al. (2019) The global syndemic of obesity, undernutrition, and climate change: the Lancet Commission Report. Lancet 393, 791–846. 30700377 10.1016/S0140-6736(18)32822-8

[48] Liberali R , Kupek E & de Assis MAA (2019) Dietary patterns and childhood obesity risk: a systematic review. Childhood Obes 16, 70–85. 10.1089/chi.2019.0059 31742427

[49] UNICEF (2023) Goal 2: Zero Hunger. https://data.unicef.org/sdgs/goal-2-zero-hunger/ (accessed 14 April 2023).

[50] Banatvala N , Akselrod S , Bovet P et al. (2023) The WHO global action plan for the prevention and control of NCDs 2013–2030. *In Noncommunicable Diseases*, pp. 234–239 [ N Banatvala , P Bovet , editors]. Abingdon: Routledge.

[51] World Health Organization (2016) *Report of the Commission on Ending Childhood Obesity*. Geneva: WHO; available at https://www.who.int/publications/i/item/9789241510066 (accessed 5 June 2022).

[52] Egger G , Swinburn B & Rossner S (2003) Dusting off the epidemiological triad: could it work with obesity? Obes Rev 4, 115–119. 12760446 10.1046/j.1467-789x.2003.00100.x

[53] Willett W (2012) Nutritional Epidemiology. Oxford: Oxford University Press.

[54] Tadjoeddin MZ (2019) Inequality and exclusion in Indonesia political economic developments in the Post-Soeharto Era. J Southeast Asian Econ 36, 284–303.

