# Supplementary material for: BMI-z score trajectories of Indonesian children and adolescents between 1993 and 2014 and associated risk factors
Source: Public Health Nutr. 2025 Jun 3;28(1):e101. doi: 10.1017/S1368980025100499 (PMC12264784; doi:10.1017/S1368980025100499)
Supplement: Widyastuti et al. supplementary material [file S1368980025100499sup001.docx]

Supplementary Material

Table S.1: Model fit for BMI-z trajectory groups in children and adolescents.

|  | BMI trajectory group | | | |
| --- | --- | --- | --- | --- |
|  | Group 1  11.7 %  n=2,344 | Group 2  28.4 %  n=7,151 | Group 3  54.3 %  n=16,503 | Group 4  5.6 %  n=1,396 |
| Mean BMIZ at baseline (intercept) | −1.91 SD | −0.01 SD | −1.0 SD | 1.16 SD |
| Average posterior probability value | 0.78 | 0.74 | 0.78 | 0.84 |
| Odds of correct classification | 26.73 | 7.08 | 9.25 | 87.31 |
| Estimated group probability | 0.09 | 0.26 | 0.60 | 0.05 |
| Proportion assigned to group according to the maximum posterior probability assignment rule | 0.12 | 0.28 | 0.54 | 0.06 |
